# Supplementary material for: ACAGT-007a, an ERK MAPK Signaling Modulator, in Combination with AKT Signaling Inhibition Induces Apoptosis in KRAS Mutant Pancreatic Cancer T3M4 and MIA-Pa-Ca-2 Cells
Source: Cells. 2022 Feb 17;11(4):702. doi: 10.3390/cells11040702 (PMC8869916; doi:10.3390/cells11040702)
Supplement: Supplementary file 1 [file cells-11-00702-s001.zip › cells-1554379-supplementary.pdf]

cells

Supplementary Material

ACAGT-007a, an ERK MAPK Signaling Modulator, in Combination with AKT Signaling Inhibition Induces Apoptosis in KRAS Mutant Pancreatic Cancer T3M4 and MIA-Pa-Ca-2 Cells

Golam Iftakhar Khandakar [1,†], Ryosuke Satoh [1,†], Teruaki Takasaki [1], Kana Fujitani [1], Genzoh Tanabe [2], Kazuko Sakai [3], Kazuto Nishio [3] and Reiko Sugiura [2,*]

1 Laboratory of Molecular Pharmacogenomics, Department of Pharmaceutical Sciences, Faculty of Pharmacy, Kindai University, Osaka 577-8502, Japan; iftakharkhandakar@outlook.com (G.I.K.); satohr@phar.kindai.ac.jp (R.S.); takasaki@phar.kindai.ac.jp (T.T.); fujitani.kana@kindai.ac.jp (K.F.)
2 Laboratory of Organic Chemistry, Department of Pharmacy, Faculty of Pharmacy, Kindai University, Osaka 577-8502, Japan; g-tanabe@phar.kindai.ac.jp
3 Department of Genome Biology, Kindai University School of Medicine, Osaka 589-8511, Japan; kasakai@med.kindai.ac.jp (K.S.); knishio@med.kindai.ac.jp (K.N.)
* Correspondence: sugiurar@phar.kindai.ac.jp; Tel.: +81-6-6730-1394
† These authors equally contributed to this work.

Citation: Khandakar, G.I.; Satoh, R.; Takasaki, T.; Fujitani, K.; Tanabe, G.; Sakai, K.; Nishio, K.; Sugiura, R. ACAGT-007a, an ERK MAPK Signaling Modulator, in Combination with AKT Signaling Inhibition Induces Apoptosis in KRAS Mutant Pancreatic Cancer T3M4 and MIA-Pa-Ca-2 Cells. *Cells* **2022**, *11*, 702. https://doi.org/10.3390/ 10.3390/cells11040702

Academic Editor: Hendrik Ungefroren

Received: 29 December 2021
Accepted: 3 February 2022
Published: 17 February 2022

Publisher's Note: MDPI stays neutral with regard to jurisdictional claims in published maps and institutional affiliations.

Copyright: © 2022 by the authors. Submitted for possible open access publication under the terms and conditions of the Creative Commons Attribution (CC BY) license (https://creativecommons.org/licenses/by/4.0/).

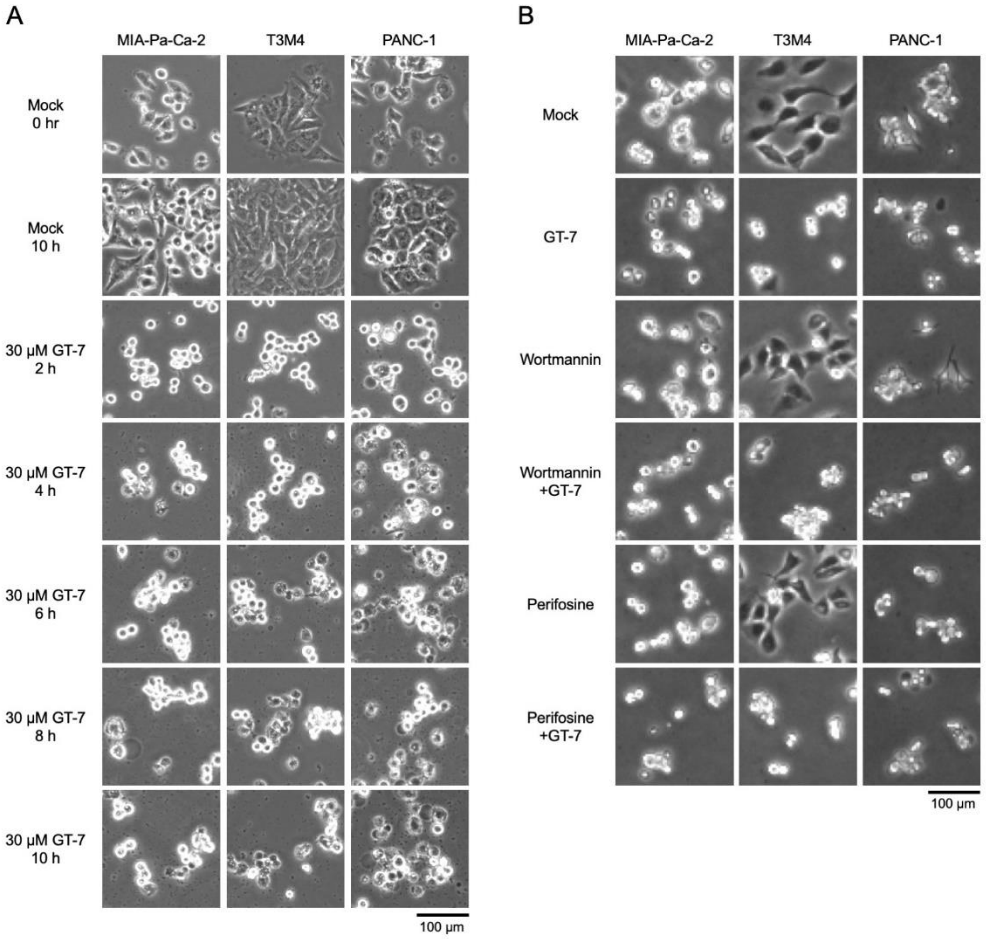

Figure S1. Phase-contrast images of PDAC cells (**A**) Time-dependent (0-10 h) phase-contrast images of the PDAC cells as indicated in Figure 3A. Bars, 100 μm. (**B**) Phase-contrast images of the PDAC cells as indicated in Figure 4A. Bars, 100 μm.

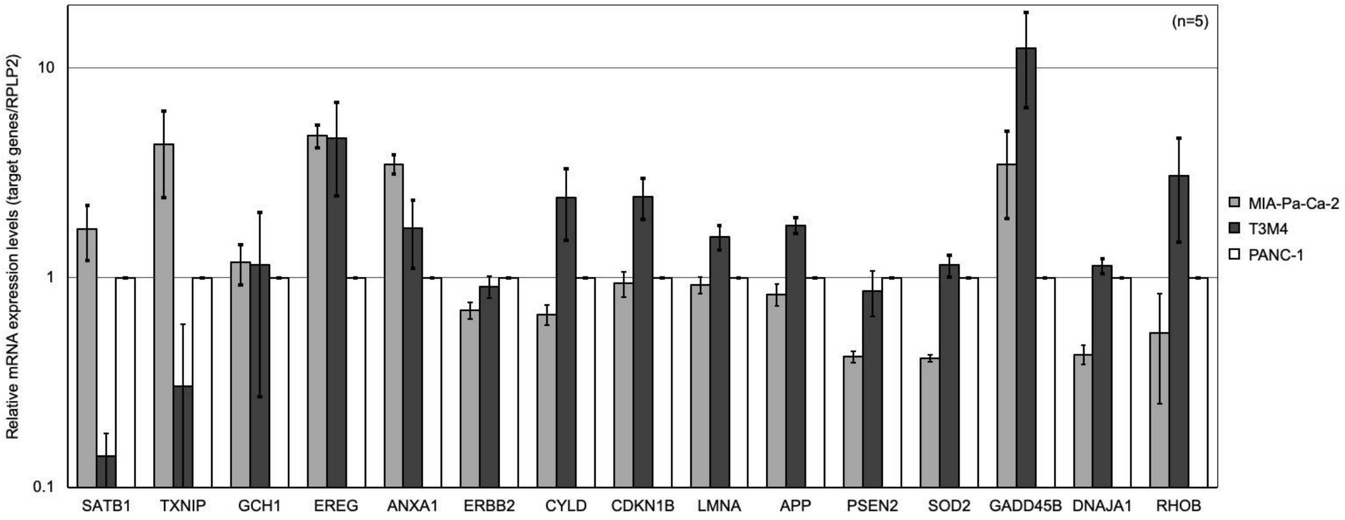

Figure S2. qRT-PCR analyses on the expression of apoptosis-related genes in PDAC cells. RPLP2 was used as a reference gene. Relative mRNA expression levels in PANC-1 cells were set as 1. The data were averaged from five independent experiments (*n* = 5). Columns, means; bars, SEM.

Table S1. List of pRT-PCR primers.

Primer Name	Sequence (5′-3′)
Slug F	CCCACACATTACCTTGTGTTTGCAA
Slug R	CAAATGCTCTGTTGCAGTGAGG
ZEB1 F	GGCAGAGAATGAGGGAGAAG
ZEB1 R	CTTCAGACACTTGCTCACTACTC
SATB1 F	AGAGCTAGCGAGGGAGAGAG
SATB1 R	CGGCTGTTGTTGTTGTGACG
TXNIP F	GGAGTGCTTGTGGAGATCGG
TXNIP R	ACGCCGCTGGTTACACTAAG
GCH1 F	GCGTACCTTCCTCAGGTGAC
GCH1 R	CTTCGAGGTCTGCGGCTAAA
EREG F	TACTGCAGGTGTGAAGTGGG
EREG R	GTGGAACCGACGACTGTGAT
ANXA1 F	AGTTCTTTGCAAGAAGGTAGAGA
ANXA1 R	CTGATCCGGGACCACCTTTG
ERBB2 F	GCAGGATATCCAGGAGGTGC
ERBB2 R	TTCAGCGGGTCTCCATTGTC
CYLD F	CAGAGAGTGTGACGCAGGAA
CYLD R	ACCTTTGTCCCCAACACCTC
CDKN1B F	AGTGTCTAACGGGAGCCCTA
CDKN1B R	CCGGGTTAACTCTTCGTGGT
LMNA F	CTACACCAGCCAACCCAGAT
LMNA R	GGTCGAAGGACAGAGACTGC
APP F	TGGAGGTACCCACTGATGGT
APP R	TGTGCATGTTCAGTCTGCCA
PSEN2 F	TGACAGTTTTGGGGAGCCTT
PSEN2 R	CTCCTCTTCCTCCAGCTCCT
SOD2 F	GCTGGAAGCCATCAAACGTG
SOD 2 R	GAAACCAAGCCAACCCCAAC
GADD45B F	AACGACATCAACATCGTGCG
GADD45B R	GTGTGAGGGTTCGTGACCAG

DNAJA1 F	GACAAAGGAGGAGAACAGGCA
DNAJA1 R	AAAGATGTCCATGGGGGAGC
RHOB F	GTGTGTCTGTTCGACTCCCC
RHOB R	AGGGATATCAAGCTCCCGCT
RPLP2 F	AAGGATCCATGCGCTACGTCG
RPLP2 R	GCGAATTCATGTCATCATCTGAC
